# Understanding the Reality of China’s Health Tourism and Consumer Demand From the Perspective of Consumers: A Cross-Sectional Study

**DOI:** 10.3389/fpsyg.2022.824328

**Published:** 2022-05-06

**Authors:** Yao Liu, Qian Lin, Dan Zhang

**Affiliations:** Institute for Hospital Management, International Graduate School, Tsinghua University, Shenzhen, China

**Keywords:** health tourism, consumer demand, attitude, promotion strategy, cognition

## Abstract

**Background:**

Travel for health reasons is booming around the world and in China. As a huge source and destination of health tourism, little is understood about the volume, characteristics, motivations, and preferences of health travelers in China. This study provides details of China’s health tourism reality and consumer demand of Chinese residents who did or will travel for health.

**Methods:**

We established a questionnaire through literature analysis and a focus group, then collected 695 responses based on an online random sampling design. Finally, 629 questionnaires (effective recovery rate was 90%) were analyzed with statistical description, binary logistic regression, and word frequency analysis to draw the reality of health tourism, explore the influential factors, and sort out suggestions.

**Results:**

In this study, 387 respondents knew of health tourism (61.53%), 446 reported interest (70.9%), and 234 had traveled for health reasons before (37.2%), with 329 occurring within China (91.4%). The top three reasons for health tourism were decompression and relaxation (116, 20%), physical examination (82, 14.1%), and health care (73, 12.6%). High costs (372, 16.3%), little disposable time (309, 13.5%), and lack of reliable professional institutions (289, 12.6%) were the main potential barriers for consumers. Professional level and quality of the institution, personal privacy, and service personnel’s attitude were the most important concerns for consumers when arranging health travel. Marital status (OR = 0.209, 95% CI = 0.085–0.514, *P* = 0.001) and attitude to health tourism (OR = 2.259, 95%CI = 1.553–3.287, *P* < 0.001) were factors for consumers’ willingness to perform health tourism. “Propaganda” was proposed most frequently by participants, followed by “service” and “price”.

**Conclusion:**

The popularity of health tourists is low although there is a huge market in China. There are also differences between willingness of previous and prospective customers with varying socio-demographic characteristics in this investigation. Overall, more diverse propaganda measures should be taken, and government policies or legal documents ought to keep pace with it. Health tourism products’ promotion, as well as supporting measures and brand, need to be emphasized.

## Introduction

Health tourism, which combines health service and tourism, has recently boomed as the social economy develops and travel demand for physical and psychological health promotion surges (Vetitnev et al., 2016). Health tourism has existed for a long time (Bauer, 2015), and was first documented in Ancient Greece. Jonathan initially created the concept of health tourism in 1987 (Goodrich and Goodrich, 1987), and it has been evolving alongside continuous research. However, a complete consensus on its definition has not been established. It is commonly accepted that health tourism is a form of tourism which comprises all natural and cultural resources, rehabilitation and sport activities, facilities, and places with services associated with the healthcare sector and tourism sector to serve people traveling for physical and mental health reasons (Huiyur, 2020). There are many categories of health tourism activities. It can be divided into obligatory or elective based on the tourist’s decision. Obligatory travel means travelers have to go outside due to the unavailability of required treatments in local place. However, elective travel usually occurs whenever they want although the service may be available in their home regions (Jones and Keith, 2006). Other research classifies health tourism according to their functions, such as medical tourism, leisure-oriented tourism, cosmetic surgery, wellness tourism, and Chinese medicine tourism (Han et al., 2018; Gongmei et al., 2021).

People continue to travel in the pursuit of health, and worldwide revenue from health tourism estimated by several authoritative global Non-Governmental Organizations (e.g., WHO, Patient without borders, Medical Tourism Association) for health tourism is large and growing. Health tourism has become the economic backbone in developed countries (Lee and Li, 2019). Nevertheless, most recent growth has been in the developing countries of Latin America, Eastern Europe, South and South-East Asia, and the Middle East (Kamassi et al., 2020). Moreover, Asia gains a large part of the international health tourism market, with many countries like Thailand, India, Malaysia, and Singapore recognized as prime destinations for healthcare seekers (Han et al., 2018; Tingfang and Shengtian, 2021). China has emerged as a popular destination for health tourists at home and abroad who come to take advantages of the abundant travel resources, high level of healthcare, and unique Chinese traditional medicine. The China Tourism Research Institute released in 2017 that the total tourism revenue for 2016 was 4.69 trillion yuan, with an increase of 13.6%.

There is also a strong interest in health tourism research. To date, a great deal of studies have been done on this area, and they reveal three notable relevant research streams. First, specific tourism activities like medical tourism (Willson et al., 2018), wellness tourism (Goodarzi et al., 2016), spa tourism, and more become the main topic. Studies tend to understand unique health tourism destinations and products from various perspectives. A study using a systematic review method indicated natural resources are essential to the development and sustainability of health tourism destinations (Pessot et al., 2021). Second, scholars explored the influencing mechanism of the health tourism industry on the associated society, politics, and economy. Results showed a positive relationship between health tourism activities and psychological, physical, and social health (Lee et al., 2020). A study found that in Italy health tourism primarily consisted of domestic travel and increased the widening of the north-south divide (Manna et al., 2020). Moreover, health tourism and health inequities shape each other in low- and middle-income countries (Ceron et al., 2019). It could likewise improve the economic growth (Cheah and Abdul-Rahim, 2018). Third, the influential factors and promotion strategies of health tourism have been studied. Korea is trying a new model by fusing medical tourism and wellness tourism (Kim and Jin, 2021). Lee and Kim (2018) found that service quality, tourism resources, and culture resources positively affected health tourists’ satisfaction. Pu et al. (2021) stated that health consciousness and subjective knowledge could predict health tourism intention, and the behavior partially mediated the function. A paper identified the key factors in China from the view of the intra-industry trade, showing that the total health expenditure per capital and the number of domestic health consumers impacted health tourism (Jiang et al., 2022). Diversifying health tourism offerings and constructing a standard framework and index system of health tourism service institutions are also suggested. Liu (2016) had built the evaluation system covering six indexes: subject, object, standard, process, team, and tool.

To survive in the continuously competitive world of the health tourism market, the Chinese government has introduced a number of promotion policies to regulate the domestic health tourism industry. For example, the National Tourism Administration officially promulgated “Standard of National Health-promotion tourism Demonstration Base,” and identified the first five bases in 2016 (Gongmei et al., 2021). And a document issued in 2017 indicated the positive role of health tourism in optimizing the allocation of medical resources, stabilizing China’s economic growth, and safeguarding the people’s livelihood. The file further pointed out that “by 2030, a relatively complete health tourism service system will be basically established and the capacity will be greatly improved.”

However, in China, health resources are relatively insufficient and unevenly distributed, which produces huge demand of seeking therapy across different provinces, cities, and even counties. The health tourism sector is still in its infancy, lacking strategic planning and systematic practice. Health tourism is basically determined by the “willingness to spend on health” of consumers (Jiang et al., 2022); tourists are considered key to business success for health-care providers in each destination (Kamassi et al., 2020). Therefore, studies from the perspective of consumers would be essential. But previous studies have analyzed health tourism from a macro view (Pocock and Phua, 2011); there is a lack of evidence-based investigation on Chinese residents knowledge and attitude toward health tourism, thus offering no scientific basis for policy proposal to boost domestic health tourism. Hence, this study aims to investigate the health tourism demand of Chinese citizens via questionnaires and explore the influential factors. Through this, we can provide practical suggestions to stimulate the health tourism industry as well as making contributions to a “Healthy China” plan.

## Materials and Methods

### Study Design and Sampling

This study adopted a mixed-method approach to fully understand Chinese health tourists’ demand. Due to the COVID-19 virus outbreak, considering the cost and time effectiveness, non-probability sampling was used (Han et al., 2018). This cross-sectional observational survey was undertaken in February and March 2021 via an online survey-related software called “Questionnaire star” that allows surveyors to produce their own questionnaires. The link generated by the software was relayed to some social networks covering people of different ages (e.g., Wechat, Weibo) (Rotonda et al., 2021; Reissmann and Lange, 2021). Participants were informed of the study purpose prior to the questions and completed it by using their phones or computers. A total of 695 questionnaires were received. After getting rid of invalid and missing cases, 629 usable returns were obtained, resulting in a response rate of 90%.

### Questionnaire Development

The questionnaire was developed through a systematic literature review and a focus review. We searched the PubMed, Web of Science, CNKI, and VANFUN databases for health tourism demand and predictors, using terms such as [health tourism, medical tourism, wellness tourism, demand, need]. Based on the existent demand and potential demand of health tourism and one open question (Lunt and Carrera, 2010; Gan and Frederick, 2015), we established a set of questions. Then these questions were all discussed and validated by five professional experts from the healthcare and international health tourism fields.

The questionnaire consisted of two parts. The first part asked basic information such as age, gender, education, marital status, health status, monthly household income, and staff position. The second part was a structured scale covering health tourists’ existent demand and potential demand (Table 1).

**TABLE 1:** Predicators for health tourists’ demand.

First level	Second level	Third level
Existent demands	Cognition of health tourism	Have known(or heard about) health tourism before
		Health tourism types known(or heard about)
		The way of getting information and knowledge about health tourism
	Health tourism experience	Have traveled for health purposes before
		Destination traveled to before
		Purposes of health tourism behavior
Potential demands	Willingness	Willingness to recommend others to take health tourism
		Willingness to take health tourism in the future
	Preference	Preference for health tourism arrangement
		Preference for health tourism types
		Preference for health tourism supporting services
		Acceptable fees
		Acceptable time spent on health tourism
	Worries	Barriers to health tourism
	Influence factors	Professional level and quality of the institution
		The confidentiality degree to the personal privacy
		The service personnel’s attitude
		Destination’s natural environment
		Supporting services(e.g., food, arrangement)
		Service project design of institution
		Transportation of destinations
		Project price of destination
		Geographical location of destination
		Oral communication of destination
		Reputation of destination
		Season features of destination
	Attitude	Attitude to developing health tourism

Existent demands focus on the cognition of health tourism and relevant experience. Questions concerning cognition included asking whether the respondent knew (or had heard about) health tourism before. Questions concerning relevant experience focused on the destination, tour arrangement (trip ways), as well as purposes of health tourism behavior. Potential demands include willingness, preference, worries, influence factors of health tourism, and attitudes toward it (Lancaster, 1966; Lee and Li, 2019). The consumer willingness was obtained through a binary single choice (“refuse” or “agree” to take part in health tourism activities if possible). Questions with respect to preference covered expected fees, duration time, and supporting service. For influence factors, a 5-point scale ranging from 1- (extremely unimportant) to 5- (extremely important) is used to measure 12 items (price, service quality, environment, transport convenience, language, and so on). In this study, the overall Cronbach’s α of the questionnaire was 0.936, the values of the Kaiser–Meyer–Olkin (KMO) measure were 0.935 (>0.9), and Bartlett’s test results were significant (*p* < 0.001), which implied satisfactory reliability and validity.

### Data Analysis

The quantitative data (the structured questionnaire) was analyzed simultaneously with the qualitative materials.

Quantitative study: The initial data was exported from “Questionnaire star,” and statistical analysis was mainly processed with SPSS 24.0 for Windows. A descriptive statistical study was performed to report participants’ demographic characteristics and the health tourism *status quo* (personal health tourism characters, existent health tourism demand, and potential health tourism demand) in China. We used means and standard deviation for numeric variables and percentages for categorical variables. The importance of health tourism influence factors was rated and sorted with an average score using a 5 Likert-type scale. Then willingness for health tourism was set as the dependent variable, using stepwise forward regression to screen variables (personal characteristics, health tourism product information), a binary logistic regression model was designed and run on the whole sample investigators to analyze influence factors. *P* < 0.05 was set as the level of statistical significance.

Qualitative study: Word frequency statistics refers to counting the number and frequency of each word in a certain text (Yunqiu and Jinkuan, 2021). In total, 214 health tourism suggestions were sent for analysis. Key words were extracted and the frequency was calculated using the word frequency analysis technology of Yi Ciyun website, so as to put forward new ideas for the promotion of health tourism.

## Results

**FIGURE 1:**
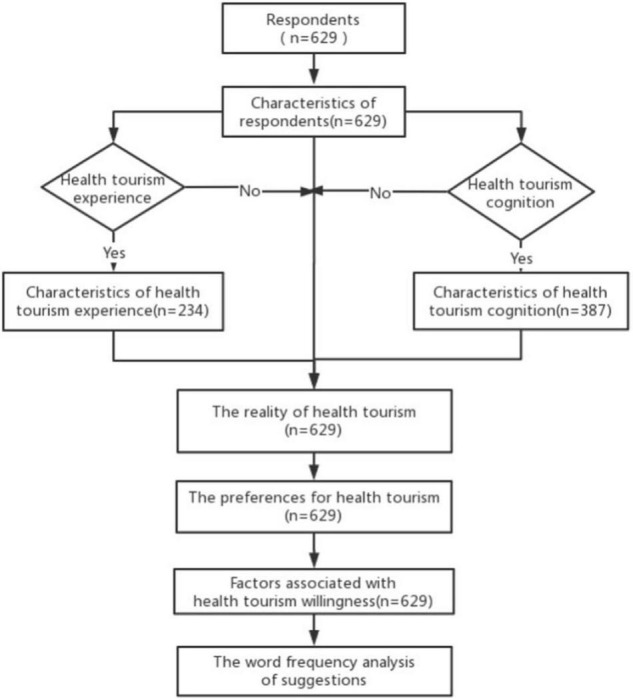
Results structure.

We divided the result into five parts as follows: Characteristics of respondents, The reality of health tourism in China, The preferences for health tourism, Factors associated with health tourism willingness, and The word frequency analysis of health tourism suggestions (Figure 1).

### Characteristics of Respondents

The 629 participants featured more young people aged between 20 and 40 years old (Table 2, 489, 77.7%), with more females than males (350, 55.6%). A majority of these people had obtained a college degree and above (465, 74%). About half were single (333, 52.9%). Many had a monthly household income between 5,001 and 10,000 yuan (176, 28%). Over half of the respondents lived in eastern China (393, 62.5%).

**TABLE 2:** Demographic characteristics of the respondents.

Variables	*n*	(%)
**Age, mean (SD), years**		
<20	24	3.8
20–29	370	58.8
30–39	119	18.9
40-49	68	10.8
≥50	48	7.6
**Gender**		
Male	279	44.4
Female	350	55.6
**Role**		
Civil servant/soldier	157	25
Technical worker	105	16.7
Commercial/service work	42	6.7
Self-employed	21	3.3
Industrial work	22	3.5
Agriculture	13	2.1
Retiree	11	1.7
Student	204	32.4
Other/unemployed	54	8.6
**Marital status**		
Single	333	52.9
Married	296	47.1
**Education level**		
Below Associate degree	70	11.1
Associate degree	94	14.9
College degree	245	39
Master degree or above	220	35
**Region**		
Eastern China	393	62.5
Central China	113	17.9
Western China	123	19.6
**Monthly household income (in yuan)**		
Nil	49	7.8
≤5000	128	20.3
5001–10000	176	28
10001–20000	162	25.8
20001-50000	83	13.2
≥50001	31	4.9

### The Reality of Health Tourism in China

Among all respondents, more than half knew of health tourism (387, 61.53%). As Table 3 shows, for these participants, the top three types of health tourism known were Leisure and fitness (229, 28.4%), Wellness tourism (206, 25.5%), and Medical tourism (194, 24%). Some of them reported how and/or where they got the information and knowledge about health tourism (195, 50.39%); travel agencies (99, 20.1%), the Internet (87, 17.6%), television/radio (67, 13.6%), and medical institution (66, 13.4) were the main sources of related information.

**TABLE 3:** Cognition of health tourism.

Variables	*n*	(%)
**Type of health tourism knew (*n* = *387, 61.53%*)**		
Medical tourism	194	24
Wellness tourism	206	25.5
Chinese medicine tourism	133	16.5
Leisure and fitness	229	28.4
Other types	24	3
Unknown	21	2.6
**Approach to information and knowledge about health tourism (*n* = *195, 50.39%*)**		
Travel agency	99	20.1
Medical institution	66	13.4
Internet	87	17.6
Television/radio	67	13.6
Newspaper/magazine	47	9.5
Relative recommendation	44	8.9
Professional recommendation	36	7.3
Propaganda brochures	21	4.3
Health tourism organization	21	4.3
Other	5	1

**FIGURE 2:**
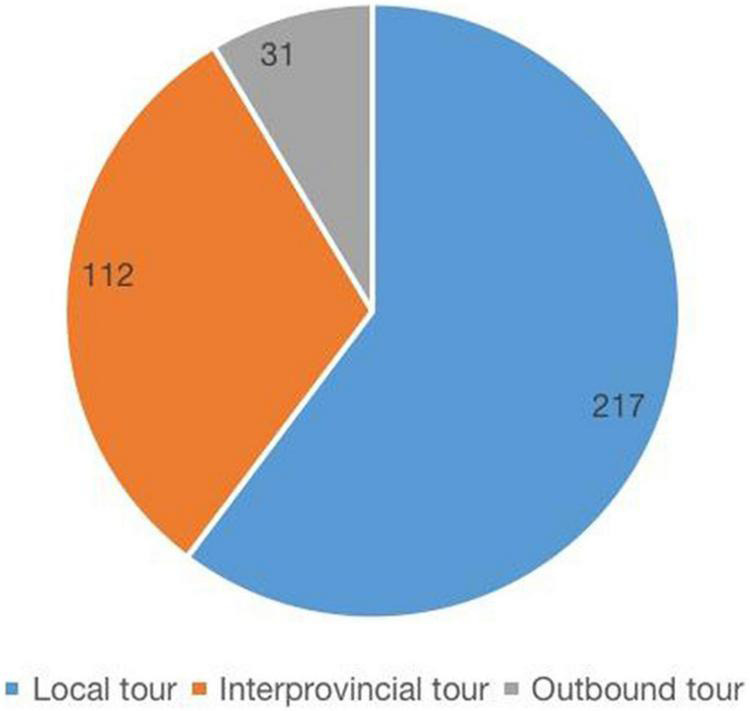
Previous health tourism destinations distribution.

**FIGURE 3:**
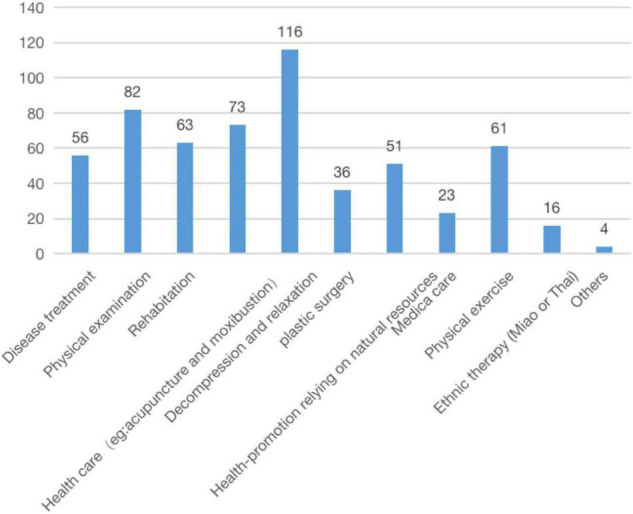
Previous health tourism purposes.

In total, 234 respondents had traveled for health reasons before, accounting for 37.2% of the whole. A majority of the health tourism occurred within China (329, 91.4%), including 217 (60.5%) within the confines of a province/an autonomous region or a municipality, and 112 (31.1%) across the provincial boundaries; 31 people (8.6%) reported going abroad. They mainly traveled to seek decompression and relaxation (116, 20%), physical examination (82, 14.1%), and health care (73, 12.6%). (Figure 2) and (Figure 3).

### The Preferences for Health Tourism

A total of 70.9% participants reported interest in health tourism (Table 4), and 71.7% preferred to go with friends and/or relatives, there is a universal demand for all kinds of service facilities, including booking service (15.1%), accommodation arrangement (16.4%), shutter service (15.3%), tourism advisory and planning (13.4%), special activities (12.7%), insurance service (10.6%), visa service (8.8%), and translation (6.9%). As for the cost, more than half would travel if the cost was less than 5000 yuan. A total of 446 residents stated that less than one week was appropriate (70.9%). Concerns including high costs (16.3%), little disposable time (13.5%), as well as lack of reliable professional institutions (12.6%) were the main barriers to potential health tourists’ decision-making.

**TABLE 4:** Health tourism preferences (*n* = *629*).

	*n*	(%)
**Willingness to health tourism**		
Very interested	204	32.4
Interested	242	38.5
Neutral	144	22.9
Not very interested	30	4.8
Not interested at all	9	1.4
**Preference for health tourism arrangement**		
Together with friends and/or relatives	451	71.7
Package tour	92	14.6
Traveling alone	72	11.4
Other	14	2.2
**Preference for health tourism supporting services**		
Booking service	430	15.1
Accommodation arrangement	468	16.4
Shutter service	436	15.3
Tourism advisory and planning	382	13.4
Special activities	362	12.7
Insurance service	302	10.6
Visa service	250	8.8
Translation	196	6.9
Other	24	0.8
**Acceptable fees (taking family as a unit, in yuan)**		
Under 1000	94	14.9
1001–3000	190	30.2
3001–5000	168	26.7
5001–10000	112	17.8
10001–20000	45	7.2
20000 and higher	20	3.2
**Acceptable time spent on health tourism(in day)**		
≦7	446	70.9
8–14	140	22.3
22–28	23	3.7
>28	20	3.2
**Barriers**		
High costs	372	16.3
Little disposable time	309	13.5
No effect	250	10.9
Harmful to health	231	10.1
Privacy disclosure	239	10.5
Distrust to institutions	187	8.2
Difficulty in communication	137	6
Shortage of professional institutions	250	10.9
Lack of credible institutions	289	12.6
Other	22	1

Furthermore, participants independently rated the importance of items they would be concerned about if they were going to take part in health tourism, and the results suggested that professional level and quality of the institution was the first concern, followed by personal privacy, and service personnel’s attitude (Table 5).

**TABLE 5:** Importance of consumers-concerning factors for health tourism (*n* = 629).

Variables	Mean	Ranking
Professional level and quality of the institution	4.38	1
The confidentiality degree to the personal privacy	4.32	2
The service personnel’s attitude	4.30	3
Destination’s natural environment	4.24	4
Supporting services(e.g., food, arrangement)	4.20	5
Service project design of institution	4.16	6
Transportation of destinations	4.13	7
Project price of destination	4.04	8
Geographical location of destination	3.97	9
Oral communication of destination	3.94	10
Reputation of destination	3.93	11
Season features of destination	3.85	12

### Factors Associated With Health Tourism Willingness

We used the binary logistic regression and identified factors associated with health tourism willingness (Table 6). We found that marital status (OR = 0.209, 95% CI = 0.085–0.514, *P* = 0.001) and attitude to health tourism (OR = 2.259, 95%CI = 1.553–3.287, *P* < 0.001) were factors of willingness to perform health tourism.

**TABLE 6:** Influence factors of health tourism willingness.

Variable	OR	95%CI		*P*-value
		Lower	Upper	
Age	1.032	0.994	1.071	0.099
Gender	1.50	0.740	3.04	0.260
Marital status	0.209	0.085	0.514	**0.001**
Family monthly income	1.269	0.919	1.753	0.148
Education	0.878	0.532	1.45	0.611
Health status	0.847	0.58	1.236	0.389
Occupation	1.022	0.899	1.162	0.738
Health tourism experience	1.657	0.714	3.845	0.239
Understanding of health tourism	1.02	0.89	1.168	0.776
Attitude to health tourism	2.259	1.553	3.287	**<0.001**
Constant	0.405			1

*Bold number is of statistical significance.*

Then, we converted each classification variable into dummy variables; marital status had a negative influence on willingness (*P* = 0.009 < 0.05), as married people are 0.341 times more likely to travel than the single population. In relation to occupation, there were significant differences only in commercial and service business. In terms of health status, better-health residents showed lower intention to participate (*P* = 0.01 < 0.05, OR = 0.095). As for attitude toward health tourism, the more people who were positive about health tourism saw it, the more likely they were to want to take part in it. Among them, the probability of people who were extremely passive and relatively passive about health tourism were 0.062 and 0.022 respectively, compared with those who were very optimistic about health tourism. In addition, health tourism experience contributed to increasing participation rate (*P* = 0.024 < 0.05). Those with health tourism experience were 2.642 times more likely to choose it than those without health tourism experience (Table 7).

**TABLE 7:** Detailed influence factors of health tourism willingness.

Variable	OR	95%CI		*P*-value
		Lower	Upper	
**Marital status**				
Single	ref.			
Married	0.341	0.152	0.763	0.009
**Occupation**				
Other/unemployed	ref.			
Commercial/service work	0.228	0.083	0.621	0.004
**Health status**				
Uncertainty	ref.			
Very healthy	0.095	0.016	0.564	0.01
**Attitude to health tourism**				
Very optimistic	ref.			
Not optimistic at all	0.062	0.01	0.391	0.003
Not very optimistic	0.022	0.008	0.066	<0.001
**Health tourism experience**				
No experience	ref.			
Having experience	2.642	1.133	6.161	0.024
constant	41.734			0

### The Word Frequency Analysis of Health Tourism Suggestions

**FIGURE 4:**
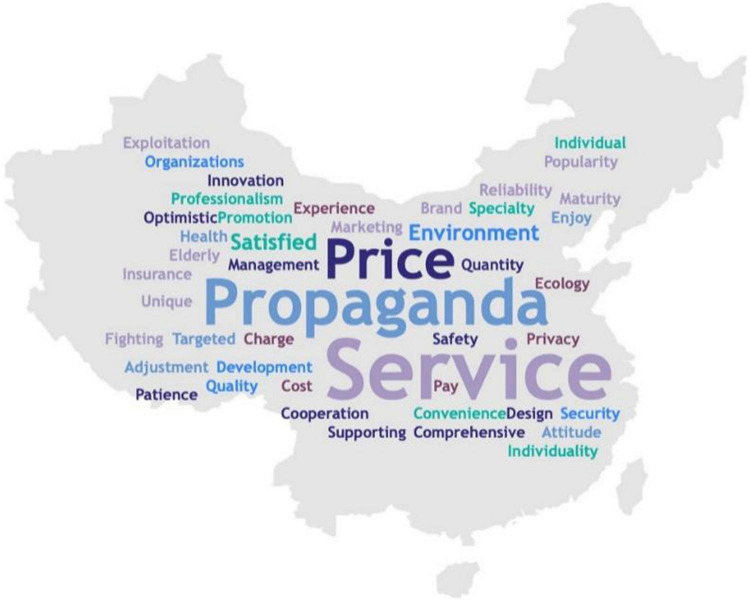
Word frequency chart of health tourism frequency.

We carried out a word frequency analysis on all suggestions collected to detect the consumers’ main focus. Results showed that “Propaganda” was proposed most frequently by participants, followed by “service” and “price” (Figure 4). Additionally, more beautiful scenic spots were expected to be built as health tourism destinations, and health tourism strategies were supposed to be individually adjusted.

## Discussion

This study was a step forward in the rarely explored area of health tourism, and provided a general overview of Chinese health tourism development. As consumer behavior has been a research trend, this survey may enormously propel the industry marketing along with promotion by analyzing and sorting influence factors from demand perspective. What’s more, the mixed method was used to thoroughly understand domestic health tourism reality.

### The Reality of Health Tourism

As a new phenomenon, health tourism has been gradually accepted in China, which is supported by our survey which saw 61.53% respondents knew about health tourism and 37.2% had experience in these activities. Compared to Koreans (79.9% respondents knew health tourism) (Han et al., 2018), the proportion of information getters and relevant experience in China were comparatively less. It is understandable that Chinese people rarely regard seeking treatment outside as an activity of tourism, thus reporting less. Moreover, that may indicate the potential to further develop domestic health tourism products. Travel agencies surprisingly became the main information provider, whereas (Musa et al., 2012) found that word of mouth obtained from close people accounted for 60.2% of health tourism understanding in Malaysia (Musa et al., 2012). It may be attributed to the prosperity of the health tourism industry in Malaysia. But in our sampling, there were a few respondents with health tourism experience before. Unlike private operators assisting travelers with the selection of doctors and hospitals as well as travel arrangements in developed countries, travel agencies usually play the advocacy role in China and the effects are not powerful enough (Johnston et al., 2011). They all imply that the health tourism market in China is nascent. The outdated policies as well as old-fashioned promotional methods may shrink its application population and hinder its expansion (Yongsheng and Tingfang, 2016), taking Hainan city for example (Yilong, 2015). Future publicity and promotion should be designed through multiple channels.

In line with our results, it has also been reported in 2018 that intra-regional tourism accounted for more than 50%. Less than 0.5% were foreign tourists (Tingfang and Shengtian, 2021) and most Italian (91%) as well as European Union citizens (92%) would like to travel within countries (Manna et al., 2020; Tingfang and Shengtian, 2021). The results encourage us to stress the significance of domestic health tourism (Han et al., 2018).

In our research, participants mostly traveled for health-promotion or relaxation, while in other Asian countries like Malaysia, Thailand, and Singapore, medical care was the pursuit (Lee, 2010; Yan, 2020). This reflects the confusion between health tourism and medical tourism. Some scholars advised to establish clear and consistent definitions of them for an increased accuracy of relevant research (Hall, 2011; Pasadilla, 2014). This is because the concept of health tourism is broader than medical tourism (Kamassi et al., 2020), and foreign scholars are used to equating them. What’s more, this may be explained by different demographic and resource structures of health tourism (Ceron et al., 2019). In Southeast Asian countries, there are inequities in service provision, whether in access to quality services or insurance (Pocock and Phua, 2011), so people have to travel abroad to get certain treatments. The level of medical technology in China is higher than the countries mentioned above (Kyu et al., 2018). And due to the aging of the population, an increasing number of Chinese people pay attention to health care and health promotion (Haiting, 2020), and would like to combine their vacation with their health tourism activities (Manna et al., 2020).

### Related Influence Factors

Residents weigh a number of factors when considering whether to begin a journey. These factors can be categorized based on the model of healthcare such as accessibility, affordability, and security. All in all, high costs, lack of disposable time, as well as lack of reliable professional institutions were the main barriers. Previous research has similarly pointed out that lower costs, shorter waiting periods, and better quality of care were the drivers of the rise of health tourism in developing countries (Ucak, 2016).

Additionally, we found marital status, health, and some occupation types associated with the willingness. Married persons show less willingness, consistent with former research (Medina-Munoz, 2013; Manna et al., 2020), because they spend lots of leisure time taking care of parents and kids. Unemployed people reported a higher probability of choosing health tourism than business or service employees, which is different from previous studies (Manna et al., 2020). This result could be linked with their increased freedom to plan and participate in travel. It reminds us that intensive job pressure in China generally consumes residents’ energy and results in less interest in health tourism, so there should be more individual marketing strategies targeted on different population groups.

It is interesting to note that health status plays a negative role on willingness. This finding probably affirms that health is one of the key destination attributes on tourists’ willingness (Lee, 2010). In contrast, Jiang et al. (2022) said Chinese health tourists are likely to visit Japan, South Korea, and Thailand for more advanced healthcare services like cosmetic surgery and anti-wrinkle treatments. Generally speaking, good health self-assessment would reduce the utilization of basic health services, subsequently reporting less participation rate. It is necessary to figure out the differences and features of health tourism demands between the domestic and overseas market. More importantly, as the Knowledge-Attitude-Practice model indicates, good cognition and a positive attitude toward something may induce relevant behaviors (Connell, 2013). It is the same as our findings that more optimistic people are interested in health tourism and more likely to participate in health tourism. Moreover, as a study reported, perceived value was a crucial predictor of tourist intentions (Habibi and Ariffin, 2019). Previous feeling during health tourism activities impacted their health perceptions (Honggang et al., 2021; Pu et al., 2021). Health benefits and health tourism knowledge attained from vacation could derive their need, which also would be spread unconsciously.

### Preferences of Chinese Health Tourists

Consistent with previous studies, tourist number, cost, and time were the driving forces to motivate health tourism (Shengtian et al., 2019). Similar to previous research (Medina-Munoz, 2013; Manna et al., 2020), the majority preferred to travel with friends or relatives autonomously. Maybe the timely support and security from fellow travelers smooths consumers psychologically and physically. Then, as Lee and Kim (2018) demonstrated, quality of medical service positively affected satisfaction of health tourism from a comprehensive research of 369 participants who had experienced health tourism. Hanefeld et al. (2014) affirmed the significance of quality standards in the health tourism industry. Our result revealed a preference for healthcare safety and quality too. However, there is an inadequate and loose inter-organizational relationship between tourism organization and famous health care organizations at present. And the profit-making of some tourism agencies is not conducive to gaining the trust of consumers. So professionalism together with consumers’ privacy are the primary and common focus (Sarwar et al., 2012). There is without a doubt a need to construct a complete and tight industrial chain including intermediary, quality of care, talent, infrastructure, and marketing for China like other hot health tourism spots in Asia (Yan, 2020).

On the whole, we systematically summarized the *status quo* and consumer demand of the health tourism industry in China. As China has a large market of tourists, some of the research results are also suitable for international health tourism promotion. It provides a chance for other health tourism destinations to preview the features and future trends of health tourism, through which we can collaboratively improve the quality of each destination.

## Conclusion

This study focuses on the domestic demand and development of health tourism in China. Firstly, a large number of people expressed willingness to conduct health tourism (70.9%), although the proportion of respondents who knew about health tourism was much lower, implying that extensive propaganda and marketing activities are needed. And diverse channels such as the internet, magazine, television, radio, and some travel agencies to spread information are needed. In addition, we could invite consumers to experience the service by themselves via relevant tickets. Apart from propaganda, government policies or legal documents ought to keep pace with the market. Especially during public health events like COVID-19, governments need to actively establish a systematic tourism program together with a health tourism monitoring system to avoid the risk of mass transmission.

Secondly, this study population with different socio-demographic characteristics showed different intentions to health tourism. Although now provincial governments are sparing no efforts to establish a comprehensive health tourism policy system covering quality supervision, medical security, and industry support, health tourism is still underdeveloped. Based on the results of the current survey, the following advice can be made. Some health tourism fees should be involved. Various and targeted health tourism routes need to be launched by travel agencies or commercial insurance institutions, such as excursion. Moreover, travel agencies are supposed to cooperate with authoritative medical institutions on the premise of taking consumer demand as the guide. It should be remembered that brand and specialty matters very much in the development. For global competition, we should create a Chinese brand, taking Chinese traditional medicine for example. For domestic competition, every region is encouraged to build a unique brand based on their characteristics, such as professional technique or beautiful scenery, to avoid repetition.

Last but not least, the importance of quality needs to be emphasized because it ranks as the most important in our test. Relevant infrastructures including transport, accommodation, and security need to be enhanced to create a comfortable residential environment. Additionally, we hope to mobilize social and association forces by setting honest and strict standards in the health tourism service, urging society to form an industry consciousness and regulation of quality.

## Limitations

This study has some limitations. The research population needs to be expanded so that the result can be generalized (Han et al., 2018). Moreover, there may be some degree of selection bias since the survey was advertised through social media (Michel et al., 2018), meaning the respondents are likely familiar with the internet (Bosnjak et al., 2013). Second, health tourism includes lots of types like traditional Chinese medicine tourism, medical tourism, and so on. There are different consumer demands due to the variance of age, income, and health status. For instance, the elderly may be interested in wellness tourism with the preference for life cultivation. Besides, due to the COVID-19 crisis, the intention to perform health tourism, especially global health tourism, may be influenced. However, we analyzed the influential factors in a general type called health tourism. Future surveys should overcome the limitation, they could choose only one type or divide into more specific classifications to get more detailed and meaningful results.

## Data Availability Statement

The raw data supporting the conclusion of this article will be made available by the authors, without undue reservation.

## Ethics Statement

The studies involving human participants were reviewed and approved by Tsinghua University. Written informed consent from the participants’ legal guardian/next of kin was not required to participate in this study in accordance with the national legislation and the institutional requirements.

## Author Contributions

DZ and YL did mainly the design and analysis of the findings. YL and QL provided a manuscript based on the data analyzed with DZ’s instruction. All authors read, revised, and approved the final manuscript.

## Conflict of Interest

The authors declare that the research was conducted in the absence of any commercial or financial relationships that could be construed as a potential conflict of interest.

## Publisher’s Note

All claims expressed in this article are solely those of the authors and do not necessarily represent those of their affiliated organizations, or those of the publisher, the editors and the reviewers. Any product that may be evaluated in this article, or claim that may be made by its manufacturer, is not guaranteed or endorsed by the publisher.
